# A Multisensor System for the Characterization of the Field Pressure in Terrain. Accuracy, Response, and Adjustments

**DOI:** 10.3390/s19183942

**Published:** 2019-09-12

**Authors:** Isabel Sicilia, Sofía Aparicio, Borja Frutos, Eduardo Muñoz, Margarita González, José Javier Anaya

**Affiliations:** 1 Eduardo Torroja Institute for Construction Science, Department of Constructions, IETcc (CSIC), 28033 Madrid, Spain; i.sicilia@ietcc.csic.es (I.S.); borjafv@ietcc.csic.es (B.F.); emunozlorenzo@gmail.com (E.M.); 2 Institute for Physical and Information Technologies “Leonardo Torres Quevedo”, Department of Acoustics and Non-destructive evaluation, ITEFI (CSIC), 28006 Madrid, Spain; m.g.hernandez@csic.es (M.G.); jj.anaya@csic.es (J.J.A.)

**Keywords:** multisensor system, differential pressures, radon transport studies

## Abstract

In different disciplines of science, the knowledge of the resulting pressures in the subsoil can help to understand physical phenomena of mass exchange between the atmosphere and the terrain. The measurement of lower differential pressures is complicated given the low range of detected values. In this paper, a multisensor system has been designed and developed to measure differential pressures in radon gas transport studies. The adequacy of this system has been proven using a purpose-built pressure chamber and an automatic motion system developed by the authors. The temporal response frequencies, the pressure values measured by the sensors, and their ability to link in series were analyzed to offer a multisensor spatial and temporal mapping. At the same time, the influence of the components required for a real deployment were studied using different tube lengths and diameters, connectors, and obstructions across the operating range of the pressure sensors. The system has also been tested for measuring differential pressures in a real model with a concrete slab above the soil and a pressure generator system below. It was found that this system is very suitable for outdoor measurements that demand a quick temporal response and accuracy.

## 1. Introduction

In different disciplines of science, the knowledge of the resulting pressures in the subsoil can help to understand physical phenomena of mass exchange between the atmosphere and the terrain. The exchange of molecular species between the external environment and the superficial soil layers mainly deals with advective processes in which the pressure gradient established between both environments plays a fundamental role.

The transport of gases in porous media can be described with mathematical models based on Navier–Stockes equations. The Darcy law described a low velocity fluid with a linear regime. Darcy terms are modified with Brinkman and Forchheimer terms to reproduce a nonlinear behavior of the gas. Other application cases are described in [1]. The description of gas transport through a soil, and the use of each model, depends mainly on pore diameter, permeability (with a high dependence of soil saturation), and the flow velocity. It allows us to establish relations between soil types and approached mathematical descriptions, as seen in [2].

Pressure in soils and its extension pore pressure are applied in disparate subjects. They are widely used in earthquakes studies, having an evolution according to wave frequencies [3]. High pressure (in the order of MPa) is applied to soil resistance improvement, like freezing processes as described in [4]. A change in soil pressure affects the soil composition. There is a relationship between changes in radon emissions and earthquake predictions, according to the analysis done by [5]. Lower pressure ranges become relevant, among others, in the study of distribution of contaminants due to air–soil exchange [6], soil aeration [7], and reduction of the infiltration of radon indoors [8,9]. The granulometric composition and biological structure of soil due to weather phenomena, like wind, influence soil pressure changes, according to experiments in [10].

The measurement of lower differential pressures between air and soil is complicated given the low range of detected values. Without external forced activity, differential pressure between outdoors (or soil) and indoors takes values from −3 to +10 Pascals [11]. The temporal inertia of the pressure response in the ground’s pores, in the absence of atmospheric pressure changes, depends fundamentally on soil porosity and it varies with depth. The knowledge of these temporal aspects is very important to evaluate the dynamic behavior of gas flows as a function of atmospheric variations [12].

According to [13], there are two bottlenecks to advance in the understanding of preferential flow in soils. The theoretical one refers to the integration of space–time relations, and the technological one refers mainly to the complexity of analysing flow patterns in situ. Knowing the dynamic behavior of subsoil flow requires sensors of great precision and high-speed responses to changes.

The purpose of this paper is to present a system of pressure data capturing, developed explicitly for the study of pressure under soil, applied to radon studies. For that purpose, a system able to detect low pressures in a range from few to several hundred Pascals, considering both positive and negative pressures, is needed. At the same time, pressure fluctuations due to weather, temperature changes, and water infiltrations requires a system able to work for a long time. The recording of simultaneous pressures in spatial dimensions, and the inclusion of temporal factor, can be applied to the study and simulation of flow patterns. An application can be seen in [14].

In this paper, an in-depth study is reported on the adequacy of the system developed by the authors to measure differential pressures. This study includes the development of laboratory experiments in order to set the system behavior. The temporal response frequencies, the pressure of the sensors, and their ability to link in series are analyzed in order to offer a multisensor spatial and temporal mapping. At the same time, the influence of the components needed for a real deployment are analyzed. Therefore, different tube lengths and diameters and the use of connectors and obstructions were tested across the operating range of the pressure sensors to analyze inertial loads due to these kinds of elements.

The system has also been tested in a real model consisting of a concrete slab, dividing soil, and the outdoor air, with a pressure generator system beneath the slab. This allows us to prove the system in a real deployment, and record real data in outer operating conditions over time.

## 2. The Multisensor System

A multisensor system has been designed and developed to study pressure differences. This system is composed of a pressure acquisition system and software based in Matlab^®^ to configure and visualize the measured data. The monitoring system was prepared for installation outdoors, with a minimum maintenance. The access to pressure supply points is done through tube connections.

### 2.1. Acquisition System of Pressure Sensors

The designed acquisition system, called PressureNet, has a segmented star architecture of 8 arms with up to 16 sensors per arm, which allows acquiring the information of a maximum of 128 sensors.

**Figure 1:**
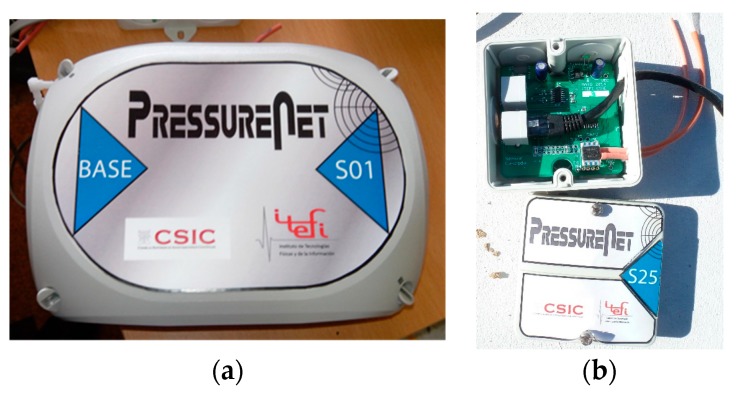
The PressureNet system developed by the authors (**a**) The multiplexer card (TMUX) card and (**b**) the modules for pressure sensors (MSP) pressure sensor.

This system consists of a multiplexer card (TMUX) and modules for pressure sensors (MSP). The differential pressure sensor, model HSCDRRD006MDSA3 purchased from HONEYWELL, supports a range of ±600 Pa with an accuracy of 3 Pa, and it was provided with SPI communication. The multiplexer card allowed us to communicate up to 8 channels of modules to a computer through a USB input–output card type LABJACK U3. Figure 1 shows some components.

For all system connections between both the TMUX and the MSP or between the MSPs of a chain, parallel ETHERNET type cables were used. The good functioning of the system was tested with cables up to 4 m long between modules. However, given the robustness of the designed system, it probably admits longer cables.

The power of the system will depend on the number of MSPs connected. The maximum consumption of each module is 40 mA. The minimum supply current of USB interface is 500 mA. Therefore, if the number of connected modules is less than 12, the power supplied by the USB connector can be used directly. Otherwise, depending on the number of sensors, one or two external sources of 5 V and 2.5 A are necessary, which are connected to the TMUX by means of micro USB type connectors.

### 2.2. Configuration and Visualization Software

**Figure 2:**
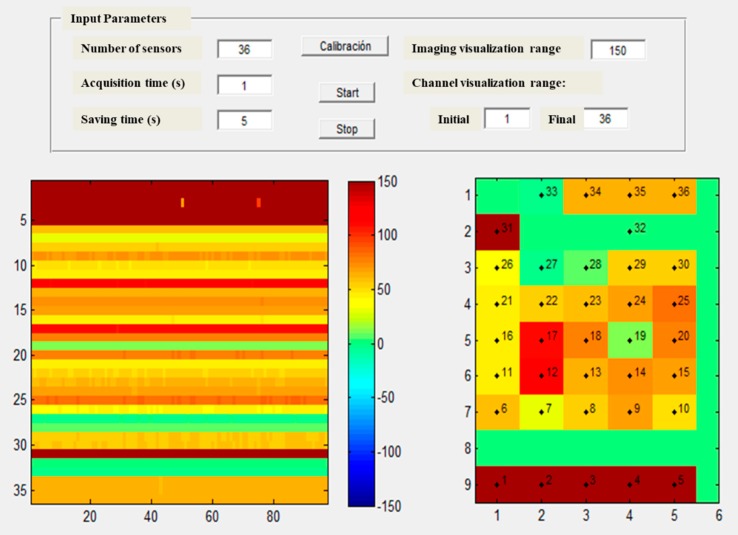
Matlab GUI of the configuration and acquisition system.

The Matlab^®^ GUI implemented to configure the system allows reading, saving, and visualization of the data measured by the sensors (Figure 2). Several input parameters have to be selected: the number of sensors, the acquisition time, how often is the average of the measured data saved, the imaging, and channel visualization range. Basically, it reads the sensor value and assigns it to a pressure map using different colors depending on the pressure value and the display range. A symmetric pressure color map has been selected with neutral central color, diverging each side to warm colors for positive values and cold colors for negative ones. At the same time, it stores the signals in a file with mat format. It also allows a reset to zero (calibration) keeping the average of 10 consecutive acquisitions and subtracting this average value to the successive pressure readings.

In the Matlab^®^ GUI, Figure 2, the pressure evolution over time is shown to the left while the right figure presents the latest instantaneous information measured by each pressure sensor.

Two assignment tables are defined within the program. The first table assigns the position of the color map to the sensor number. The second table assigns the channel and number assigned to the MSP to the sensor number. If any of these assignments are changed, it is necessary to change these tables.

## 3. Pressure System Testing

The pressure system was tested at the laboratory with a purpose-built pressure chamber. This chamber, described in Section 3.1., provides a steady pressure and is able to develop continuous or stepwise movement. In order to define the operating parameters, the usual working components, like tubes in different diameters and connectors, incidental blockages, and temporal system response in all the cases, were proven.

### 3.1. Testing System Based in a Pressure Chamber

**Figure 3:**
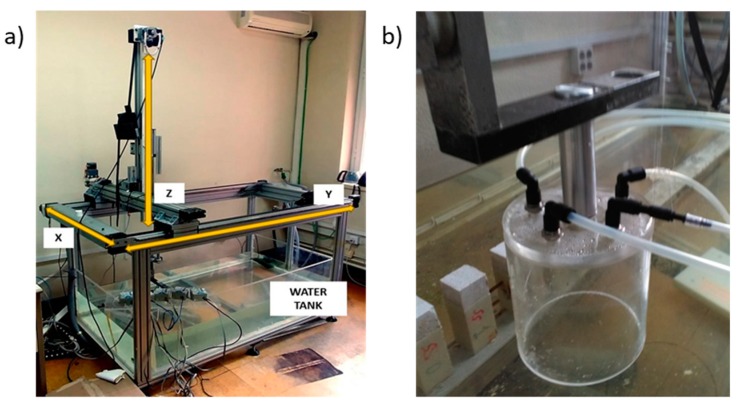
(**a**) Automatic motion system and (**b**) air pressure chamber.

A mechanical assembly was designed to provide the same pressure conditions at each experimental phase. This system consists of an air pressure chamber, a water surface, and an automatic motion system, as shown in Figure 3.

The automatic motion system is an ultrasonic imaging system. It consists of a water tank and a three-axis Cartesian robot that allows it to move the end of a support arm between two points of space (X, Y, Z), limited by the maximum displacement allowed in linear guides that are, respectively, 0.6 m, 1.2 m, and 0.7 m. Through the use of stepper motors and a suitable control card, a linear displacement can be made between any two points of this space with high precision in its position, speed, and acceleration. Putting the pressure chamber at the end of the arm and making an automatic movement in the direction of the *Z* axis allows us to vary, in a controlled and dynamic way, the pressure of the chamber by immersion in the water tank.

**Figure 4:**
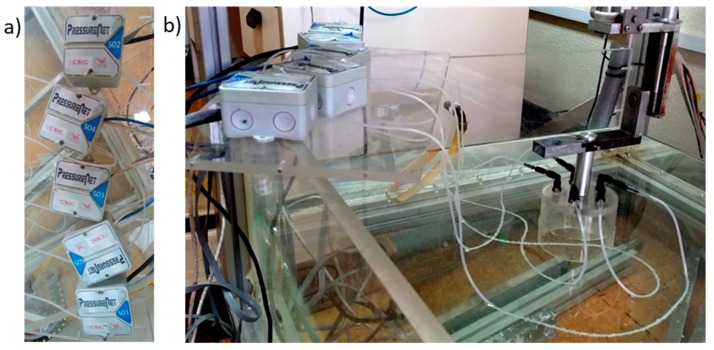
(**a**) Five pressure sensors used in the experiment and (**b**) pressure sensors connected to the air pressure chamber.

Figure 4a shows the acquisition system used in testing consisting of one chain with 5 modules connected to a LabJack U3 PressureNet, as described Section 2.1. Modules are numbered from one to five. They are connected at the top of the chamber system using push-in fitting connectors displayed in Figure 3b.

**Figure 5:**
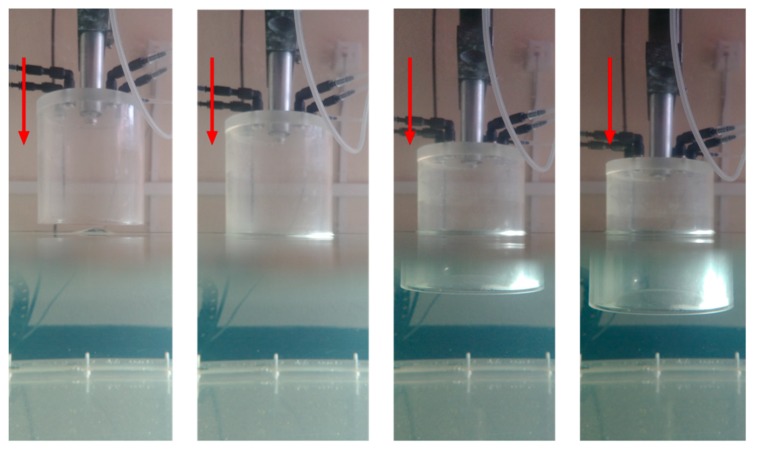
Movement of the air pressure chamber during the experiments.

At the beginning of each test, the water surface was just aligned with the bottom of the pressure chamber. Sensor data were measured for each sensor every 5 mm of descent in the pressure chamber, and until the pressure value measured with the sensor is saturated, see Figure 5. 

**Figure 6:**
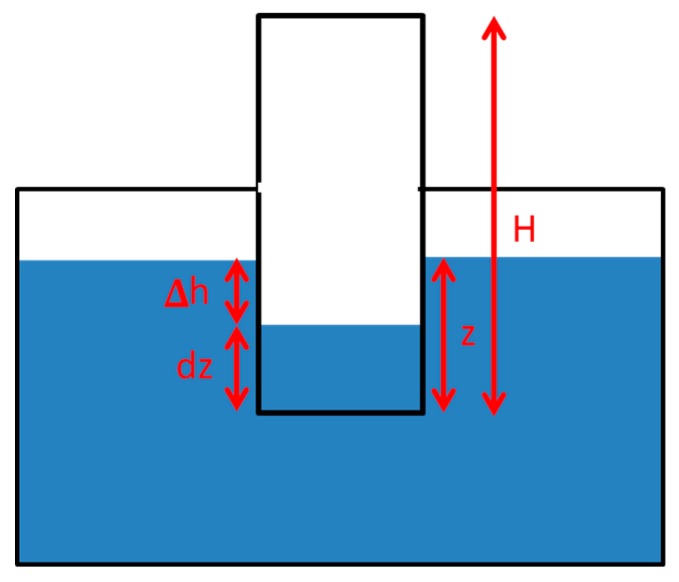
Scheme of the air pressure chamber inside the water.

All the experiments were achieved under laboratory conditions at steady temperature, so we can consider that there were no significant pressure changes due to temperature oscillations. The pressure P in the chamber as a function of the depth that the sensor is introduced into the water, z, is:

P(z) = ρ*∆h + P_a_ (1)

where ρ is the specific weight of the water, ∆h = z − dz (as depicted in Figure 6), and P_a_ the atmospheric pressure.

Considering that the temperature is constant, according to Boyle’s law, dz can be obtained from equation:

P_a_*H = (ρ*(z − dz) + P_a_)*(H − dz) (2)

where H is the total height of the chamber as shown in Figure 6.

It can be verified that for H = 10 cm and normal values of pressure and temperature, dz is approximately 1% of z, so the differential pressure is:

P(z) − P_a_ = 0.99*ρ*z. (3)

### 3.2. Elements Analyzed: Tubes, Connectors, and Obstructions

To verify the proper functioning of the system presented in Section 2, different tube sections and lengths were also tested with different numbers of connectors and obstructions.

**Figure 7:**
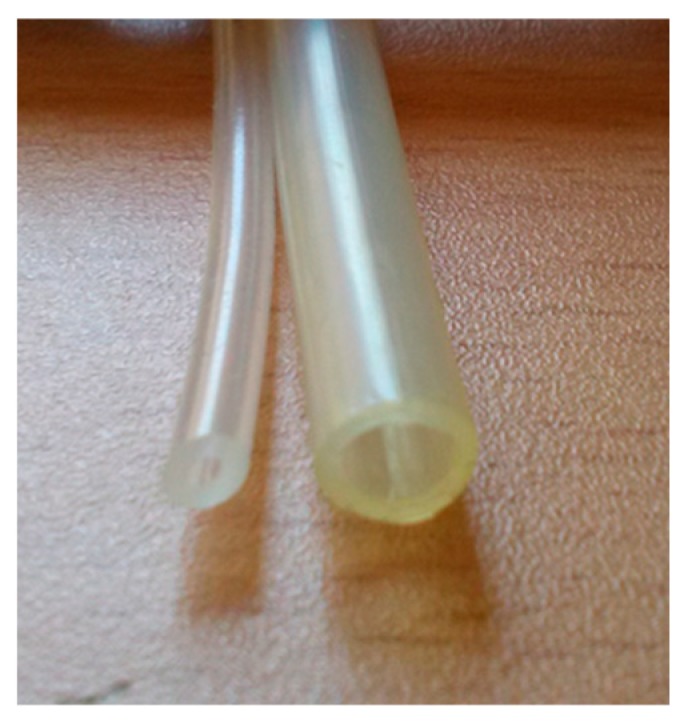
Tubes with small and large section used in the experiments.

The simplest connection system, shown in Figure 7, consists of a silicone tube treated with peroxide of 1 mm of inner diameter and 3 mm of outer diameter (small section tubes). This kind of tube is widely used in medical and pharmaceutical applications and can be directly connected to the pressure sensor (MSP). For open-air installations exposed to harsh weather conditions, larger cross-section softpolyurethane tubes are recommended, since they have more stability and allow easy mounting. The softpolyurethane tube has inner and outer diameters of 4 and 6 mm, respectively (Figure 7).

**Figure 8:**
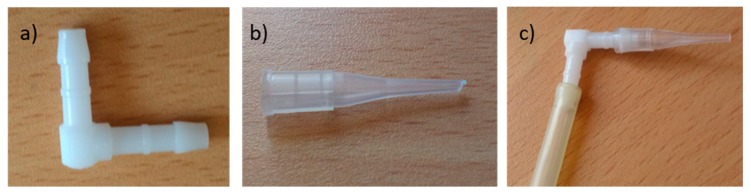
(**a**) Elbow connector, (**b**) cone-shaped connector, and (**c**) large section tube with both connectors.

The connection of these tubes with the pressure sensor (MSP) is made through an elbow with a cone-shaped connector, as can be seen in Figure 8. This mounting type may be used for most real applications. 

The elbow considered is made of polyoximethilene. It has a hose inner diameter of 4 mm male coupler section and snap fit.

**Figure 9:**
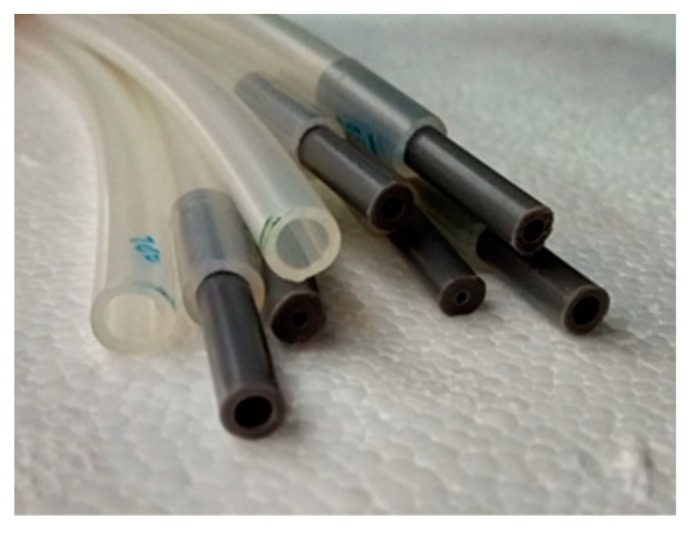
Small plastic reducers to simulate blockages.

Figure 9 shows obstructions analyzed with small pieces, especially designed for our purpose. These pieces have a length of 3 cm and an outer diameter of 4 mm. The inner wall of each piece was perforated with a diameter of 1, 2, and 3 mm to simulate different blockage levels. The pieces snap in a 4 mm tube.

## 4. Testing Experiments

The acquisition system presented in Section 2 was tested to establish its accuracy and to define its operating parameters. Therefore, different experiments were performed to study:

- Static pressure drop due to tube characteristics: diameter, length, connectors, and incidental blockages.
- Temporary response.
- Repeatability.

Test software was programmed to register measurements of five sensors simultaneously every second. In the static test, the arithmetic mean of 10 measurements is considered, in order to avoid temporal shortcomings. Sensor data were measured for each sensor every 5 mm of descent in the pressure chamber. These pressure levels vary from a 0 Pa pressure, just before the beginning of the experiment, to the pressure that causes saturation, near 600 Pa. The cycle of pressures consisted of 15 steps of 5 mm (pressure levels).

### 4.1. Static Pressure Drop Test

The objective of this test is to determine how pressure measurements can be influenced by the tube properties: tube diameter, tube length, addition of connectors or direction changes, and incidental blockages. The test includes the analysis with different pressure values, from 0 Pa to pressure sensor saturation, around 600 Pa.

For the static test, pressure in chamber is measured once it is steady. Four cases were conducted to analyze the pressure drop:

- Case (A) Loss of pressure due to tube length using a tube with 1 mm inner diameter. Pressure measurements were made using tube lengths from 15 to 400 cm.
- Case (B) Loss of pressure due to tube length using a tube with 4 mm inner diameter. Pressure measurements were made using tube lengths from 15 to 400 cm.
- Case (C) Loss of pressure due to elbow connectors. Pressure measurements were made using tubes with 1, 2, 3, and 4 elbow connectors, equally distributed across a 2 m tube of 4 mm inner diameter, and a control tube without connectors.
- Case (D) Loss of pressure due to blockages: dust, gravel, or small entries of water, over a tube of 4 mm inner diameter. Blockages were simulated using small plastic reducers, with an inner free diameter of 1, 2, and 3 mm, see Figure 9. Pressure measurements were made using 3 tubes with a single blockage over a 2 m tube, a tube with the 3 kinds of blockages at the same time, and a control tube without blockages.

The pressure chamber supports five tubing positions. Tests for Case A and Case B (loss due to tube length) were done in two phases. The first phase used tubes from 15 to 50 cm and the second one used tubes from 100 to 400 cm. Tube length, connectors, and blockages were placed as described in Table 1.

### 4.2. Temporary Response

Temporary response test includes pressure variation according to velocity, in order to detect delays in pressure register. The same cases described in Section 4.1 were analyzed considering as control case the one performed with equal tubes. The pressure chamber undertakes continuous motion from the water surface to 80 mm depth. Downward velocity varies from 10 to 40 mm/s.

Through the automatic movement system we placed the lower edge of the camera on the surface of the water. We raised it slowly until the water surface tension expired and consequently, at that moment, the camera reached atmospheric pressure. Then, we submerged the camera in the water tank by a downward movement of 80 mm and the automated movement started. It was programmed to perform up and down cycles indefinitely with 80 mm of vertical displacement. The automatic system allowed us to modify two parameters, maximum speed and acceleration. Its movement was evenly accelerated until it reached the maximum speed, then it became a uniform movement until it started to decelerate to stop at 80 mm from the starting point. The direction of movement was inverted and the acceleration ramp started again to carry out the same movement in the opposite direction. The speeds and accelerations used in the experiment are shown in Table 1.

### 4.3. Repeatability

The pressure chamber is able to provide steady pressure for five modules at the same time, and to develop the same downward movement. For each case, three equal series were done, in order to study repeatability. Each series was composed of 15 measurements at different pressure values. In the same way, some of the tubes used in Cases A and B had the same length, in order to prove data stability regardless of module. Each case was repeated as shown:

Static cases A and B:
  - For lengths of 15, 25, 100, and 400 cm: six repetitions of 15 pressure levels each.
  - For lengths of 50 and 200 cm: three repetitions of 15 pressure levels each.
Static cases C and D:
  - For each tube combination: three repetitions of 15 pressure levels each.
Temporary cases:
  - 400 measures, corresponding to 6 (lower velocity) or 16 (higher velocity) complete cycles.

All these series are summarized in Table 2.

## 5. Testing Results

### 5.1. Pressure Drop Results

**Figure 10:**
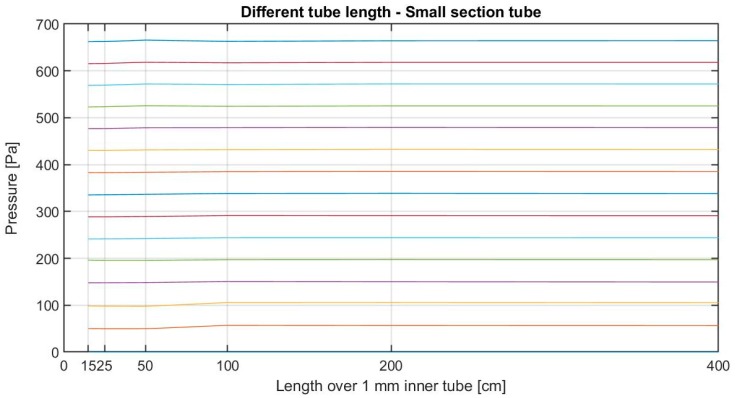
Static pressure measurement using a 1 mm inner tube of different lengths.

Pressure measurements were not affected by changes in tube length, tube thickness, connectors, or obstructions. Pressure differences were transmitted as long as the air connection channel exists, regardless of its length or shape. An example of this can be seen in Figure 10, where all the tube configurations led to similar results. This absence of leaks is due to the use of air as working fluid and its conditions of environmental density and viscosity at low velocity.

According to Equation (4), and taking into account that under pressure of one atmosphere and temperature of 20 °C, ρ = 9.8 kN/m^3^, the expected regression coefficient is 9.7 Pa/mm. Table 3 shows the results for each sequence of differential pressure oscillation, analyzed using a simple linear regression model. In this way, we can analyze and compare the system behavior for different pressure ranges. As can be seen in Table 3, the pressure difference between real data and theoretical assessment is from 1.5% to 5.5%, with real measurements being slightly lower.

### 5.2. Repeatability of the Results

System stability is proven with the repetition of each experiment three times. The pressure chamber automatically changed. In order to give comparable results, all experiments were based over a control tube of 2 m for static cases, and 1 m for temporary ones.

The data can be compared using a linear model and its standard deviation. As can be seen in Table 3, the standard deviation for all the experiments, with at least three sets and three levels at each set, is very close to one. Results are consistent with the linear model proposed. There were no substantial variations due to the tube configuration tested.

### 5.3. Temporary Response Results

**Figure 11:**
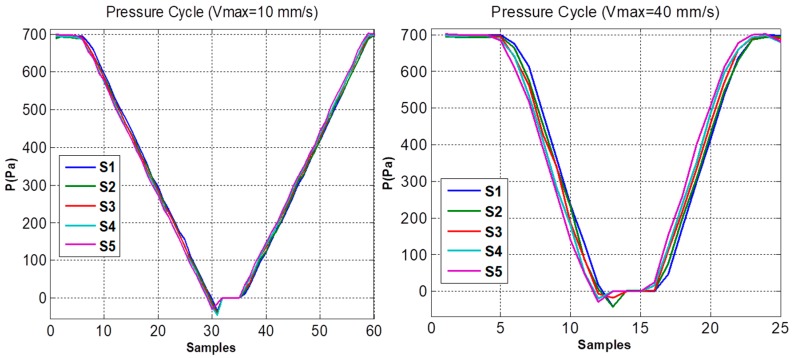
Pressure measurement performed during a cycle when the velocity is 10 and 40 mm/s.

By means of the procedure described in Section 4.2, the pressure in the chamber can be changed at constant velocity and the dynamic effect that the different tubes produce on the measurement of pressure in the system can be compared, as shown in Figure 11.

The system digitizes the pressure sequentially. Therefore, the pressure value P_n_(k) measured in each of the n sensors is:

P_n_(k) = P_on_(((k − 1)*N + n − 1)*T_s_) (4)

where k is the measurement number, N the total number of sensors, n the sensor number, T_s_ the sampling period, and P_on_ the pressure at the sensor input. Considering that there are no pressure losses in the different tubes, as it was obtained with the static experiments, we can establish the following expression for the pressure at the sensor entrance:

P_on_(t) = P_i_(t − t_n_) (5)

where Pi is the pressure in the chamber and t_n_ is a delay that occurs in the pressure transmission due to the structure, section, and length of the tubes.

Considering constant velocity, we obtain that:

z = V*(t − t_0_) (6)

where t_0_ is the time when the constant speed starts. Thus, from the Equation (4) we get that

P_i_(t) = 0.99*V*ρ*(t − t_0_) + P_a_ (7)

and substituting C_v_ = 0.99 * V_m_ * ρ for each velocity used, we obtain that:

P_n_(k) = C_v_*((n + (k − 1)N)*T_s_ − t_0_ − t_n_) + P_a_. (8)

Subtracting the first sensor pressure value to each sensor pressure value and considering the direction of rise or fall of the system, we obtained an independent expression of k:

P_n_ − P_1_=C_v_*((n − 1)*T_s_ − (t_n_ − t_1_)) in the top zones (9)

P_n_ − P_1_= −C_v_*((n − 1)*T_s_ − (t_n_ − t_1_)) in the bottom zones. (10)

**Figure 12:**
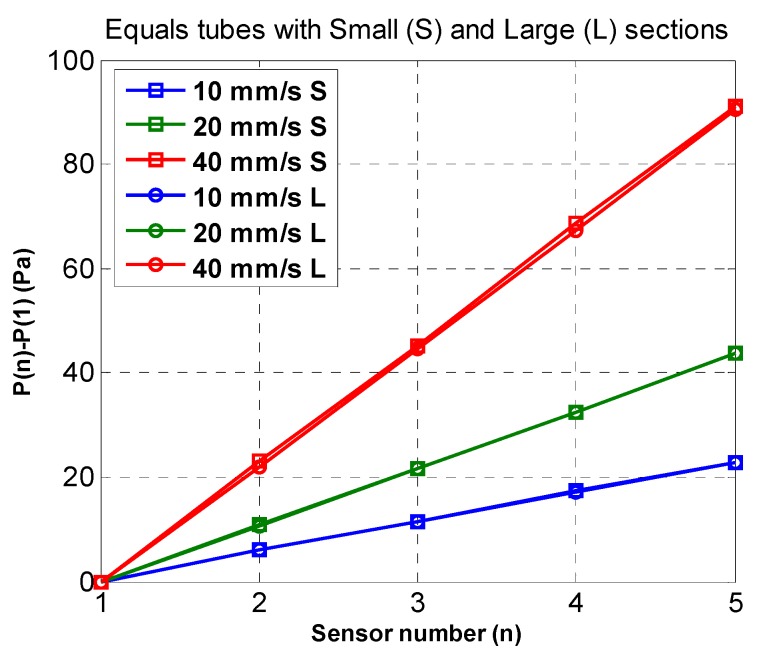
Pressure measurement with respect to the first sensor using equal tubes of different sections (small and large) and different acquisition velocities.

The average of the absolute value of the k acquisitions where the speed is constant was used to decrease the error. In the experiment with equal tubes of different diameter, see Figure 12, it can be verified that:

P_v_ = P_n_ − P_1_ = C_v_*((n − 1)*T_s_). (11)

Adjusting the previous results to a straight line and taking into account that under pressure of 1 atmosphere and temperature of 20 °C, ρ = 9.8 kN/m^3^, then T_s_ = 56.6 ± 0.8 ms.

Replacing these values in Equations (9) and (10), the time delays of the pressure transmission with respect to the first sensor can be computed depending on the velocity.

t_n_ − t_1_ = 0.057·(n − 1) − 0.000103·(P_n_ − P_1_)/V. (12)

The average for the three velocities used and the standard deviations are shown in Table 4.

**Figure 13:**
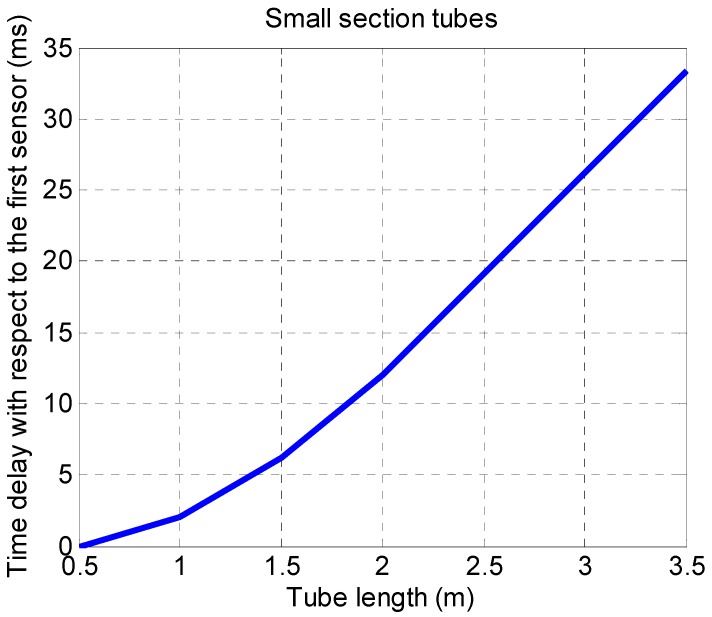
Relationship between the length of the tube and the delay with respect to the first sensor.

There are no significant detectable variations in the transmission times due to the different structures, except for the small section tubes. In this case, as can be seen in Figure 13, the relationship between the length of the tube and the delay with respect to the first sensor is practically linear.

## 6. Application and Use: Real Scale Model

**Figure 14:**
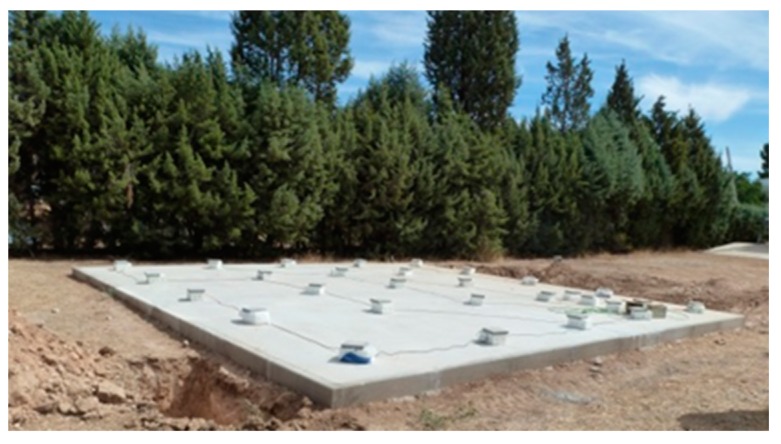
Experimental slab in Arganda del Rey (Madrid)—Spain.

The system presented here is set to register differential pressure data in soils over time, allowing us to know soil pressure regarding temperature, pressure, rain, and other weather effects. Application fields are soil fertilization, analysis of permeability, control, and elimination of natural and artificial gas contaminants, like radon. In order to test the system on a real application field, a concrete slab was built over a pressurized air generator system in the soil beneath. This allowed us to provide positive or negative pressurized air into a line under the slab. As shown in Figure 14, works were made in open field, so the system can be used to estimate the movements and flow of underground air. Inner pressure transmission depends on permeability and soil characteristics.

**Figure 15:**
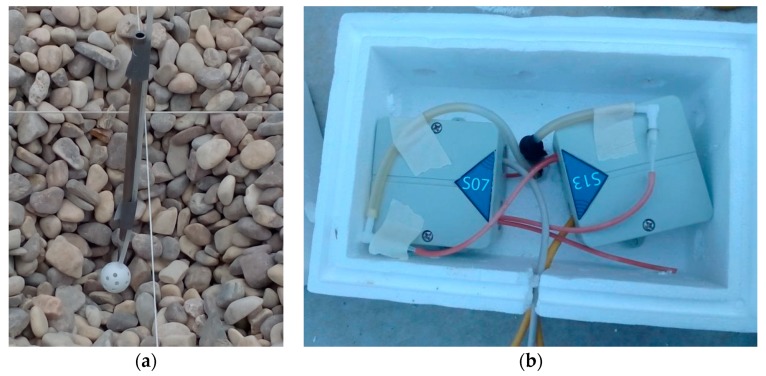
(**a**) Tube insertion on ground and gravel. (**b**) Double sensor connection on the experimental slab.

**Figure 16:**
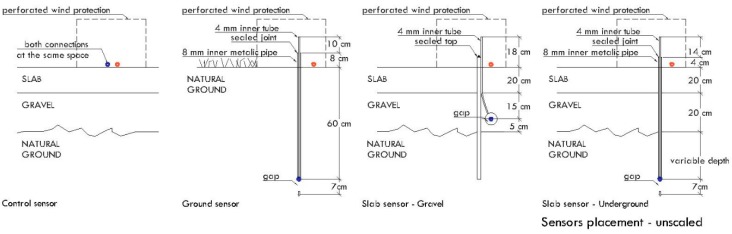
Tube positions for field test experiments.

The concrete slab, with dimensions of 8 m × 8 m × 0.2 m (length × wide × height), was built over a base of gravel 4/20 according to Spanish standard. The slab was provided with a metal pipes grid and a section of 4 mm inner tube (Figure 15a). This helped us to insert the end of the sensor just to the appropriate depth. Connections with pressure sensors were done through a 15 cm length × 1 mm inner tube diameter, a cone-shaped connector, and a single elbow connector system attached to a 4 mm inner tube (Figure 15b). Tube length was adapted to the depth of caption point (Figure 16) and varied between 45 and 150 cm in order to determine soil pressure gradient in depth. According to test experiments, this configuration has no substantial impact on measured values. All the joints and pipes were carefully sealed in order to reduce pressure loses.

**Figure 17:**
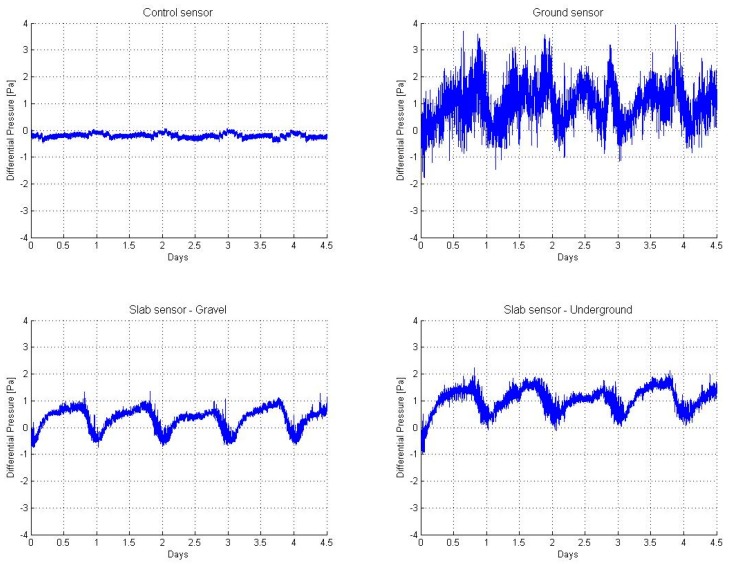
Behavior of natural differential pressure between atmosphere and soil over time.

The data were registered in different situations. First of all, the behavior of natural pressures over a long time period was computed, comparing open-air and underground pressures. Figure 17 shows an example of the data registered during 4.5 days. The pressure patterns were replicated in the same way with other sensors, in similar positions under slab.

After that, the movement of air and differences of pressures were set using the pressure generator system. The pressure was measured at two different planes: in the middle of the gravel and in natural soil under the slab surface. Sensors were placed at different points of the slab and pressure generator system. Data were recorded at the highest speed: one measure per second. The data stored can be a single one or a geometrical mean of the measures registered. The system is designed to operate and record data continuously along diverse periods of time, from one second (highest record speed) to several years.

**Figure 18:**
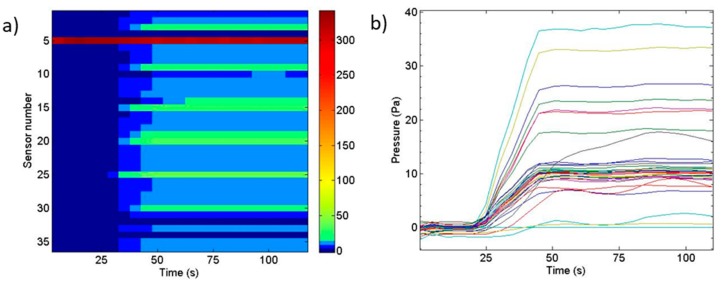
Evolution of the pressure value over time for each sensor using a colormap in (**a**) and plotted in (**b**).

Figure 18 shows an example of the pressure transmissions at underground level using large section tubes with the pressure generator system activated. In this figure, the evolution of the pressure value over time for each sensor is shown using a colormap in a) and plotted in b). In Figure 18a, red color is for pressures around 300 Pa and blue color for 0 Pa. Sensor 5 was saturated because it was placed in the pressure generator. In these pictures, the delays between sensors can be detected and it can be confirmed that they are not due to the system characteristics, as has been studied in the previous section. Therefore, it can be concluded that the dynamic terrain behavior is not altered by the tubes used in the measurement system.

According to realized experiments, there is a significant difference in pressure distribution through the different soil compositions. Pressure distribution at gravel level is produced homogeneously, as long as the pressure distribution at underground level depends on the soil characteristics. A more compact soil gives a higher gradient of pressure, reducing its scope, while a less compact and permeable soil gives a less pronounced (even non-existent) gradient, with a highest scope and lower maximum pressure. The left hand diagram in Figure 18 shows pressure evolution over time that in this case, with a regular and sustained air impelled, remains constant.

Tests are realized by supplying different pressures and under diverse weather conditions, giving as a result similar pressure distribution. Despite system stability, the use of protection against wind and rain is recommended. The system is robust and supports environmental temperatures, but sensors are very sensitive to wind pressure and depression.

In order to form relationships between pressure transmission and soil permeability, the data were compared with a laboratory experiment. PressureNet system was applied to a test apparatus filled with different granular fill material types [15]. Material types included natural soil from the experimental slab place and the gravel 4/20 used under slab. Pressures were measured in different heights along a 1 m material column, in order to stablish gas permeability. Pressures were tested in ranges between 20 and 600 Pa.

## 7. Conclusions

In this paper, a multisensor system has been designed and developed to study pressure differences to be applied in radon gas transport studies. The adequacy of this system was proven using a purpose-built pressure chamber, developed by the authors, and an automatic motion system. The temporal response frequencies, the pressure of the sensors, and their ability to link in series were analyzed in order to offer a multisensor spatial and temporal mapping. At the same time, the influence of the components required for a real deployment were studied. Therefore, different tube lengths and diameters and the use of connectors and obstructions were tested across the operating range of the pressure sensors.

According to the pressure drop test results described in this paper, data registered accuracy was up to 5% with respect to the calculated theoretical pressure. At the same time, there was no significant difference in the variation of tube length between 15 and 400 cm. Likewise, there was no difference when the number of connectors between tubes increased up to 4.

Tubes were tested with two different inner diameters: 1 mm and 4 mm. Punctual reduction of inner dimensions in the 4 mm tube, such as those corresponding to a dust or other element entry, did not affect the system measurements. However, a complete tube sealing, like a water entry, may be avoided due to the blockage of pressure transmission.

Temporary response was also analyzed using different velocities of the automatic system from 10 to 40 mm/s, equivalent to variations of pressure from 98 to 588 Pa/s. The system digitizes the pressure sequentially, so increasing the sensor number increases the transmission time. This delay is computer dependent of a few milliseconds. There was no significant time variation due to use of different connection configurations: tube length until 3.5 m, inner diameter variation from 1 mm to 4 mm, inclusion of 1 to 4 connectors or obstructions.

System stability was proven with the repetition of each experiment three times. In this experiment, the pressure measurement remained stable at the different cases’ steps.

In order to test the system on a real deployment, a concrete slab was built over a pressurized air generator system. The application in the real scale model verified good behavior in outdoor conditions. PressureNet system has been proven to be very adequate for the measurement of pressures outdoor, having a quick temporal response and accuracy.

## Figures and Tables

**Table 1:** Velocity and acceleration of the automatic system used in the experiment.

Maximum Velocity (mm/s)	Acceleration (mm/s^2^)	Ramp Time (s)	Average Velocity (mm/s)
10	50	8.50	9.41
20	50	4.70	17.02
40	50	3.11	25.72

**Table 2:** Experimental cases and series.

**Static Response Test**						
**Case A: Length Variation in a Tube with an Inner Diameter of 1 mm**						
Series	Pressure Levels	Sensor 1	Sensor 2	Sensor 3	Sensor 4	Sensor 5
		(cm)	(cm)	(cm)	(cm)	(cm)
A1 A2 A3	15	15	15	25	25	50
A4 A5 A6	15	100	100	200	400	400
**Case B: Length Variation in a Tube with an Inner Diameter of 4 mm**						
Series	Pressure Levels	Sensor 1	Sensor 2	Sensor 3	Sensor 4	Sensor 5
		(cm)	(cm)	(cm)	(cm)	(cm)
B1 B2 B3	15	15	15	25	25	50
B4 B5 B6	15	100	100	200	400	400
**Case C: Variation of Number of Connectors in a 2 m Tube of 4 mm Inner Diameter**						
Series	Pressure Levels	Sensor 1	Sensor 2	Sensor 3	Sensor 4	Sensor 5
		Number	Number	Number	Number	Number
C1 C2 C3	15	0	1	2	3	4
**Case D: Obstructions (Free Inside Diameter) in a 2 m Tube of 4 mm Inner Diameter**						
Series	Pressure Levels	Sensor 1	Sensor 2	Sensor 3	Sensor 4	Sensor 5
		(mm)	(mm)	(mm)	(mm)	(mm)
D1 D2 D3	15	4	3	2	1	3 + 2 + 1
**Temporary Response Test**						
**Case A: Length Variation in a Tube with an Inner Diameter of 1 mm**						
Velocity	Pressure Levels	Sensor 1	Sensor 2	Sensor 3	Sensor 4	Sensor 5
(mm/s)		(cm)	(cm)	(cm)	(cm)	(cm)
10, 20, 40	15	50	100	150	200	350
**Case B: Length Variation in a Tube with an Inner Diameter of 4 mm**						
Velocity	Pressure Levels	Sensor 1	Sensor 2	Sensor 3	Sensor 4	Sensor 5
(mm/s)		(cm)	(cm)	(cm)	(cm)	(cm)
10, 20, 40	15	50	100	150	200	350
**Case C: Variation of Number of Connectors in a 2 m Tube of 4 mm Inner Diameter**						
Velocity	Pressure Levels	Sensor 1	Sensor 2	Sensor 3	Sensor 4	Sensor 5
(mm/s)		Number	Number	Number	Number	Number
10, 20, 40	15	0	1	2	3	4
**Case D: Obstructions (Free Inside Diameter) in a 2 m Tube of 4 mm Inner Diameter**						
Velocity	Pressure Levels	Sensor 1	Sensor 2	Sensor 3	Sensor 4	Sensor 5
(mm/s)		(mm)	(mm)	(mm)	(mm)	(mm)
10, 20, 40	15	4	3	2	1	3 + 2 + 1

**Table 3:** Linear regression coefficients for each experiment.

**Length over 1 mm Inner Tube**				
**Tube Length**	**Series**	**Pressure Levels**	**Linear Model Coefficient (95% Confidence Bounds)**	**R-Square**
15 cm	6	15	9.427 (9.373, 9.48)	0.9993
25 cm	6	15	9.437 (9.386, 9.487)	0.9994
50 cm	3	15	9.483 (9.413, 9.552)	0.9994
1 m	6	15	9.385 (9.318, 9.452)	0.9989
2 m	3	15	9.406 (9.309, 9.503)	0.9989
4m	6	15	9.411 (9.345, 9.477)	0.9989
**Length over 4 mm Inner Tube**				
**Tube Length**	**Series**	**Pressure Levels**	**Linear Model Coefficient (95% Confidence Bounds)**	**R-Square**
15 cm	6	15	9.45 (9.394, 9.506)	0.9992
25 cm	6	15	9.444 (9.389, 9.5)	0.9992
50 cm	3	15	9.49 (9.41, 9.569)	0.9993
1 m	6	15	9.55 (9.51, 9.589)	0.9996
2 m	3	15	9.536 (9.478, 9.595)	0.9996
4m	6	15	9.526 (9.485, 9.567)	0.9996
**Number of Connectors over 200 cm 4 mm Inner Tube**				
**Number Connectors**	**Series**	**Pressure Levels**	**Linear Model Coefficient (95% Confidence Bounds)**	**R-Square**
0	3	15	9.233 (9.113, 9.352)	0.9982
1	3	15	9.192 (9.073, 9.311)	0.9982
2	3	15	9.224 (9.108, 9.339)	0.9983
3	3	15	9.2 (9.084, 9.315)	0.9983
4	3	15	9.255 89.142, 9.368)	0.9984
**Obstructions over 200 cm 4 mm Inner Tube**				
**Free Diameter**	**Series**	**Pressure Levels**	**Linear Model Coefficient (95% Confidence Bounds)**	**R-Square**
4 mm	3	15	9.551 (9.481, 9.62)	0.9994
3 mm	3	15	9.509 (9.444, 9.574)	0.9995
2 mm	3	15	9.54 (9.473, 9.607)	0.9995
1mm	3	15	9.519 (9.453, 9.585)	0.9995
3 + 2 + 1 mm	3	15	9.573 (9.51, 9.636)	0.9995

**Table 4:** Average of time delays (s) in the pressure transmission with respect to the first sensor for the 3 velocities used and standard deviations.

	Sensor 1		Sensor 2		Sensor 3		Sensor 4		Sensor 5	
	t_1_ − t_1_	σ	t_2_ − t_1_	σ	t_3_ − t_1_	σ	t_4_ − t_1_	σ	t_5_ − t_1_	σ
Equal	0	0	−1	3	−1	2	−1	5	−3	5
Elbows	0	0	−1	4	−1	3	−1	4	0	4
Small section	0	0	2	2	6	5	12	5	33	6
Large section	0	0	−1	0	−2	2	−3	3	−1	3
Blockages	0	0	−2	1	0	2	0	2	1	3
